# Supplementary material for: Assessing the geographic specificity of pH prediction by classification and regression trees
Source: PLoS One. 2021 Aug 11;16(8):e0255119. doi: 10.1371/journal.pone.0255119 (PMC8357141; doi:10.1371/journal.pone.0255119)
Supplement: Attachment — Submitted filename: Response to Reviewers.docx [file pone.0255119.s003.docx]

**RESPONSE TO EDITOR:**

EDITOR COMMENT: *The submitted manuscript has some merit but I totally agree with both reviewers that this work needs some major revisions before being considered to be published in PLON ONE. I'm mainly concern about the short description of the state of the art (Introduction) and the methods section. refer 2 highlighted this point in the revisions, Other aspect is that I've some difficulty to the highly the scientific novelty of this work and the future applications. The authors should clearly highlight this aspect.*

**RESPONSE:** **Thank you for coordinating the review process. We have revised the**

**manuscript and addressed Reviewer’s comments. To address your recommendations, we have elaborated on the importance of our research in the Discussion section.**

**General Comments:**

**COMMENT**: Please ensure that your manuscript meets PLOS ONE's style requirements, including those for file naming.

**RESPONSE: We have checked that all formatting and naming requirements have been met.**

**COMMENT:** We note that Figures 1 and 3 in your submission contain map images which may be copyrighted. We require you to either (a) present written permission from the copyright holder to publish these figures specifically under the CC BY 4.0 license, or (b) remove the figures from your submission:

**RESPONSE: We have obtained permission from the copyright holder, ZeeMaps, to publish their images. A PLOS ONE Content Permission Form has been uploaded.**

**RESPONSE TO REVIEWER 1:**

REVIEWER COMMENT: *The manuscript is well written, the introduction is short but contain all the necessary information for the current study. The goals of the study are simple and well designated, very understandable and in agreement with what the authors want to understand beyond what is already known. Material and methods section is well designed with necessary detail. The results are well presented and divided into appropriate key points. The discussion section is in accordance with the presented goals and results from the study and the conclusions are well described, according to the discussion of the obtained results and aligned with the hypotheses presented in the goals and tested with the results. With a short revision (indicated below in the specific comments and considered as minor revision) in order to improve the manuscript, I recommend this research for publication.*

**RESPONSE: We thank the reviewer for their kind words. We have revised our manuscript according to their recommendations below.**

REVIEWER COMMENT: *Line 103: Caption of Fig. 1: where is “Created” should be “created”.*

**RESPONSE: We have revised the caption of Fig. 1 to read “Study area. Points indicate state parks where samples were collected. Created with ZeeMaps.”**

REVIEWER COMMENT: *Line 107: dH20 is deionized water? If yes, please indicate in the text; “…vigorously shaken…” during which time? And how? Please indicate!*

**RESPONSE: We specified this line to read “deionized H2O.” We replaced the phrasing of “vigorously shaken” with a more specific description: “The mixture was vigorously inverted and rotated before being let to sit for 10 minutes…”**

REVIEWER COMMENT: *Please describe each of the parameters in the equation (1).*

**RESPONSE: We added definitions of y,** $\hat{\boldsymbol{y}}$**, and** $\bar{\boldsymbol{y}}$**, as well as an in-text description of the equation.**

REVIEWER COMMENT: *Line 148: Where is (2), (44) indicate as (2, 44). Are both references right?*

**RESPONSE: We redid notation to clearly distinguish between references to a citation and references to an equation. References to an equation are now labeled and in brackets.**

REVIEWER COMMENT: *Lines 167-173: you should indicate the meaning of all abbreviations used in the table and not only of those discussed. You can add this information in the end of the table as notes.*

**RESPONSE: We added all abbreviations in the subsection Topographic and climatic data.**

REVIEWER COMMENT: *Line 177: According to the table 3 the percentage of 26.6 is actually 26.4 (see table 3). please correct accordingly in the text.*

**RESPONSE: We re-constructed Table 3 with data from Spearman correlations rather than Pearson correlations. We performed this step to accommodate reviewer 2’s methodological recommendations. Updated values in the table match those in the text, but the specific sentence identified here was removed because it is not appropriate to square the Spearman Rho.**

REVIEWER COMMENT: *Line 205: Since the manuscript only have 3 figures I think that you can use this figure in S2 in the manuscript too, as fig. 4.*

**RESPONSE: We have added figure S2 in to the manuscript as figure 4.**

**RESPONSE TO REVIEWER 2:**

REVIEWER COMMENT: *The manuscript “Assessing the geographic specificity of pH prediction by classification and regression trees” by Egelberg et al. intends to evaluate the suitability of machine learning on the prediction of pH soil, by replicating the approach of Zhang et al. (2019) using CART model and by complementing it with random forest models.*

*The manuscript has a good structure and it is easy to read. However, I do find some weaknesses that should be revised to improve the paper. My major concern is related with the overall lack of detail in the manuscript. The hypothesis should be clearly indicated.*

**RESPONSE: See subsection Aim of the current study in the Introduction for our hypotheses.**

*The discussion in particular lacks an integration of data obtained, whereas the authors provide general information without discussing their data.*

**RESPONSE: Data from the Results was incorporated into the Discussion to support each claim made.**

*Other comments are also indicated. I suggest the authors a major revision of the manuscript.*

**RESPONSE: We thank the reviewer for their effort and attention to detail. We have revised our manuscript according to their recommendations below.**

REVIEWER COMMENT: *Lines 91-93 should precede the aims. Can the authors give more detail on the accuracy of RF prediction?*

**RESPONSE: We believe the logical flow of the paper is better maintained by stating the study objectives prior to our expectations regarding those objectives. As such, we have kept lines 91-93 before the aims. This decision aligns with the recommendation of Schober et al. in “Clear Study Aims and Hypotheses in a Research Paper.”**

**However, we updated our hypotheses to provide a more specific estimate regarding the accuracy of RF prediction. Because we believed that the RF would be more accurate than the CART, and that our CART would demonstrate an accuracy in our region similar to Zhang et al.’s 6.9% RRMSE in their region, we hypothesized that our RF would yield a % RRMSE of below 6.9.**

REVIEWER COMMENT: *Is there any information that could be added to describe pH in the soils sampled?*

**RESPONSE: We added a statement describing the observed pH range in our study region.**

REVIEWER COMMENT: *Can the authors provide a reference for the procedure adopted?*

**RESPONSE: We added a reference from which we derived our protocol.**

REVIEWER COMMENT: *The use of pH strips does not provide sufficient accuracy for the measurements, which could benefit the model approach used by the authors.*

**RESPONSE: We updated the description of our methodology with additional details and citations to support its robustness. Specifically, we added that we used four-squared pH test strips for pH testing. Previous literature has found these types of test strips to exhibit greater than 95% positive and negative predictive power and greater than 90% sensitivity and specificity in moderately acidic pH. Considering that our observed pH range falls within this moderately acidic category, four-squared plastic test strips were appropriate in the current context.**

REVIEWER COMMENT: *Table 1 is merely informative and in part replicates tables 2 and 3. It could be in supporting information since it does not provide substantial information relevant to study.*

**RESPONSE: We moved Table 1 to supporting information.**

REVIEWER COMMENT: *Did the authors check the normality of the data?*

**RESPONSE: We did not check for normality; however, all sample sizes exceed 30 samples. By the central limit theorem, this implies that the sampling distribution of our sample means for all factors is approximately normal and t-tests are appropriate. We applied Levene’s test for homogeneity of variance found variances to differ between samples. As such, we applied a two-tailed Welch’s t-test to our data. We added citations to our manuscript to support our analysis.**

REVIEWER COMMENT: *Lines 167-168: to assess ‘the strength of the linear association between variables and soil pH’ using Pearson correlation the authors have to indicate if variables did present normal distribution.*

**RESPONSE: We recalculated Pearson correlations with the Spearman correlation, a rank-based method that does not assume normality. This recalculation altered the strength and significance of the relationship between some factors and soil pH, but our initial conclusions remain largely intact. Changes have been clearly noted in the revised manuscript. We thank the reviewer for bringing this assumption of the Pearson correlation to our attention and have added a citation that discusses the appropriate use of correlation coefficients to support our analysis and as a resource for future readers.**

**Further, though this was not requested by the reviewer, in our recalculation of correlational significance we adjusted our p-values with the Bonferroni correction. Because we performed multiple statistical tests on different characteristics of the same samples, the global probability of a type I error is inflated. To counter this, we multiplied p-values by the number of tests performed as suggested by Bonferroni. This is mathematically equivalent to dividing the probability of a type I error (α) by the number of tests. We added references to support this decision. We did not perform this step in the original manuscript because the authors were made aware of the Bonferroni correction after our initial submission.**

REVIEWER COMMENT: *Lines 219-221: This is a conclusion drawn from your work. Please, revise the start of your discussion.*

**RESPONSE:** **We agree that this is a conclusion drawn from our work, but we dispute that this is problematic to include in this section. We believe it is important to address the accuracy of our hypotheses upfront in the Discussion and we believe that our conclusions are relevant to our discussion of the results.**

**To clarify the flow of our Discussion, we have split it into subsections. Each of these subsections interprets a portion of our results in the context of our initial hypotheses. We have also expanded the interpretation of our results in the context of previous literature.**
