# Supplementary material for: Assessing the geographic specificity of pH prediction by classification and regression trees
Source: PLoS One. 2021 Aug 11;16(8):e0255119. doi: 10.1371/journal.pone.0255119 (PMC8357141; doi:10.1371/journal.pone.0255119)
Supplement: Attachment — Submitted filename: Response to Reviewers.docx [file pone.0255119.s004.docx]

**RESPONSE TO EDITOR:**

EDITOR COMMENT: *After careful consideration, we feel that it has merit but does not fully meet PLOS ONE’s publication criteria as it currently stands. Therefore, we invite you to submit a revised version of the manuscript that addresses the points raised during the review process. After a careful reading of the reviewers comments (mainly reviewer 2) i tend to agree that the manuscript needs some clarification before being accepted for publication in PLOS ONE. While reviewer 2 comment 1 and 4 are a question of style I tend to corroborate both comments, suggesting a revision accordingly. Comments 2 and 3 from the same reviewer are more delicate and needs a clear clarification. I agree with the reviewer that the normality of the data should be checked. Also the Pearson and Spearman correlations issue should be clarified.*

**RESPONSE:** **Thank you for your comments. We have addressed your recommendations with our responses to Reviewer 2’s input.**

**GENERAL COMMENTS:**

COMMENT: *Please review your reference list to ensure that it is complete and correct. If you have cited papers that have been retracted, please include the rationale for doing so in the manuscript text, or remove these references and replace them with relevant current references. Any changes to the reference list should be mentioned in the rebuttal letter that accompanies your revised manuscript. If you need to cite a retracted article, indicate the article’s retracted status in the References list and also include a citation and full reference for the retraction notice.*

**RESPONSE: We have reviewed our references and can ensure that it is complete and correct. There are no retracted papers included in our references.**

**RESPONSE TO REVIEWER 1:**

REVIEWER COMMENT: *Taking into account that the authors made all the changes proposed and that the manuscript was improved with the help of the comments of both reviewers, I considered that this paper can be published in the present form.*

**RESPONSE: We thank the reviewer for their time and consideration.**

**RESPONSE TO REVIEWER 2:**

REVIEWER COMMENT: *The manuscript “Assessing the geographic specificity of pH prediction by classification and regression trees” by Egelberg et al. was revised according to the previous reviewer 2’ comments. However, some details need to be clarified. Consequently, I suggest the author to revise the manuscript in some critical issues, in particular points 1, 2 and 4, as follows.*

**RESPONSE: We thank the reviewer for their additional input and address each recommendation below.**

REVIEWER COMMENT: *I strongly disagree with the logical argument provided by the authors. Contrarily to what is stated by the authors, one can only postulate objectives after elaborating a hypothesis, which is completely different from a hypothesis elaborated after the definition of objectives. Therefore, lines 93-97 of the revised manuscript should precede the definition of the aims of the study. Ultimately, the response given by the authors corroborates my observation.*

**RESPONSE: We reformatted our objectives and hypothesis so that statement of the objectives follows statement of the hypotheses. We trust the reviewer’s judgement that this improves clarity.**

REVIEWER COMMENT: *The CLT has some disadvantages and is a common misconception if we take a large number of samples their distribution will be (close to) normal. Not everything is a mean. Therefore, it should be used very carefully when dealing with environmental samples. As an example, if you consider two vertical profiles, e.g. for pH, between 1 to 10-cm depth and one presents the highest value at the surface (1-cm) and other presents the same highest value at 6-cm, their distributions are completely different despite having the same average value. This is even more complicated when such variable depends on other parameters of the soils, as it is the case of this work when looking into a spatial distribution. Thus, normality should be checked using the appropriate statistical test and indicated in the Methods for the sake of the reader withdraw its own conclusions.*

**RESPONSE: To test for normality, we plotted histograms of predicted and actual pH data with the hist() function in R. CART and random forest-predicted pH values appear normally distributed; however, sampled pH and Zhang et al.’s predicted pH values do not.**

**To air on the side of caution and avoid an improper analysis, we recalculated significance with non-parametric Wilcoxon Rank Sum tests that do not assume a normal sampling distribution of sample means. The recalculation of significance did not alter our results or conclusions.**

REVIEWER COMMENT: *Please be aware that Pearson and Spearman correlations have different assumptions, so I assume the authors meant to say they have recalculated correlations.*

**RESPONSE: We agree that the Pearson and Spearman correlations measure fundamentally different phenomena and intended to state that we replaced calculations of the Pearson correlation with calculations of the Spearman correlation in our analysis. We altered language in our paper to account for these divergent assumptions. Specifically, we removed R^(2) values, replaced r values produced by the Pearson correlation with rho values produced by the Spearman correlation, and reworded our statistical analysis section to emphasize that Spearman correlations measure the strength of monotonic relationships (as opposed to Pearson correlations, which are only valid for linear relationships).**

REVIEWER COMMENT: *I do not understand or agree with this logic, as was earlier expresses for the objectives and hypothesis. One can discuss their findings and subsequently draw the conclusions but to discuss results based in conclusions is not logical and scientifically incorrect.*

**RESPONSE: We have reworded the introduction to our discussion in accordance with the reviewers’ comments.**
